# Point-of-Care Serum Proenkephalin as an Early Predictor of Mortality in Patients Presenting to the Emergency Department with Septic Shock

**DOI:** 10.3390/biomedicines12051004

**Published:** 2024-05-02

**Authors:** Christos Verras, Sofia Bezati, Vasiliki Bistola, Ioannis Ventoulis, Dionysis Matsiras, Sotirios Tsiodras, John Parissis, Effie Polyzogopoulou

**Affiliations:** 1 University Emergency Department, Attikon University Hospital, 12462 Athens, Greece; sofiabezati@gmail.com (S.B.); mats.dionysis@gmail.com (D.M.); jparissis@yahoo.com (J.P.); effiepol@med.uoa.gr (E.P.); 2 2nd Cardiology Department, Attikon University Hospital, 12462 Athens, Greece; vasobistola@yahoo.com; 3 Department of Occupational Therapy, University of Western Macedonia, 50200 Ptolemaida, Greece; iventoulis@uowm.gr; 4 4th Department of Internal Medicine, Attikon University Hospital, 12462 Athens, Greece; sotirios.tsiodras@gmail.com

**Keywords:** septic shock, point-of-care, biomarker, proenkephalin, emergency department

## Abstract

Background: The aim of the present study is to investigate the prognostic utility of point-of-care (POC)-measured proenkephalin (PENK), a novel biomarker, in terms of predicting in-hospital mortality in patients presenting to the emergency department (ED) with septic shock. Methods: Bedside PENK was measured in consecutive patients presenting to the ED with septic shock according to the Sepsis-3 clinical criteria. The association of PENK with inflammatory and routine biomarkers, and its role as a predictor of in-hospital mortality, was examined. Results: Sixty-one patients with septic shock [53% females, median age 83 years (IQR 71–88)] were evaluated. Median (IQR) values of creatinine, plasma lactate, soluble urokinase plasminogen activator receptor (SuPAR), procalcitonin and PENK were 1.7 (1.0–2.9) mg/dL, 3.6 (2.1–6.8) mmol/L, 13.1 (10.0–21.4) ng/mL, 2.06 (0.84–3.49) ng/mL, and 205 (129–425) pmol/L, respectively. LogPENK significantly correlated with LogLactate (rho = 0.369, *p* = 0.004), LogCreatinine (rho = 0.537, *p* < 0.001), LogProcalcitonin (rho = 0.557, *p* < 0.001), and LogSuPAR (rho = 0.327, *p* = 0.011). During hospitalization, 39/61 (64%) patients died. In a multivariable logistic regression model, logPENK was an independent predictor of in-hospital mortality (OR 11.9, 95% CI: 1.7–84.6, *p* = 0.013). Conclusion: POC PENK levels measured upon presentation to the ED strongly correlated with metabolic, renal and inflammatory biomarkers, and may serve as a predictor of in-hospital mortality in patients with septic shock.

## 1. Introduction

Sepsis and septic shock are critical conditions with high morbidity and mortality rates, and pose a significant burden on healthcare systems worldwide, thus necessitating prompt and effective management strategies [1]. According to the Third International Consensus definition, “sepsis is a life threatening clinical syndrome characterized by organ dysfunction induced by dysregulated host response to infection”. Septic shock refers to the subgroup of patients with sepsis accompanied by severe cardiovascular, cellular and metabolic compromise, as evidenced by the need for vasopressors to achieve a mean arterial pressure (MAP) ≥65 mmHg and by hyperlactemia (lactate > 2 mmol/L) [1]. In the most recent analysis for the Global Burden of Disease that evaluated data from 195 countries, the global incidence of sepsis was estimated at 48.9 million (95% uncertainty interval [UI] 38.9–62.9) cases of sepsis and 11.0 million (10.1–12.0) sepsis-related deaths in 2017, with diarrheic and lower respiratory infections being the leading causes [2]. Regarding hospital-treated sepsis, a recent meta-analysis reported worldwide estimates of sepsis and severe sepsis up to 31.5 million and 19.4 million cases, respectively, leading to 5.3 million deaths annually [3]. In another study published by the Intensive Care over Nations (ICON) audit, the most prevalent sources of sepsis were respiratory tract and abdominal infections. Notably, 51.6% of patients had more than one microorganism identified, and most common infectious organisms were Gram-negative (67.1%) and Gram-positive (49.8%) bacteria, while fungi and viruses represented less prevalent infectious agents with a prevalence of 12.9% and 2.9%, respectively [4]. Although sepsis had been mostly attributed to Gram-negative bacteria in the past, recent data point towards a rise of Gram-positive bacterial, fungal and viral infections [5,6].

Inflammatory response in the context of sepsis is governed by several pathophysiological mechanisms that lead to multi-organ dysfunction and death. Excessive cytokine production and complement activation result in tissue hypoxia, cytopathic injury, mitochondrial dysfunction, coagulopathy and derangements in the vascular endothelium [7,8]. The Sequential Organ Failure Assessment (SOFA) scoring system is mostly used to identify the severity of organ insult considering certain respiratory, circulatory, coagulation, hepatic, neurological and renal parameters [9]. Risk factors for the development of sepsis include older age, higher Simplified Acute Physiology II Score (SAPS II), cancer, advanced chronic heart failure, cirrhosis, the need for invasive ventilation or renal replacement therapy, and infection with *Acinetobacter* spp. [4]. Furthermore, older age, high procalcitonin and lactate levels, higher SOFA and Acute Physiology And Chronic Health Evaluation-II (APACHE-II) scores, lactate clearance rate, combined fungal infection and Gram-negative bacterial infection, acute kidney injury (AKI), development of acute respiratory distress syndrome, and disseminated intravascular coagulation, as well as patient comorbidities and immunosuppression, have been identified as prognostic factors for poor outcomes [10,11]. Early identification of septic patients who are at risk of poor outcomes is crucial in order to optimize care of individuals presenting to the emergency department (ED) with sepsis or septic shock. AKI is one of the most frequent complications in septic patients while, at the same time, sepsis represents one of the leading causes of AKI in critically ill patients. Sepsis-induced AKI is associated with high mortality and increased healthcare costs [12,13].

Recently, biomarkers have emerged as useful tools for risk stratification, prognostication, and guidance of therapeutic decisions in septic patients. Among numerous biomarkers studied in this context, serum proenkephalin (PENK) has received attention for its potential prognostic value in predicting clinical outcomes in critically ill patients. PENK is a member of the enkephalin family, a group of endogenous opioid peptides which are expressed in a variety of tissues and act by binding mainly to δ (delta)-opioid receptors, thereby activating them. Enkephalins are involved in various physiological processes, such as modulation of pain perception, cellular growth, preconditioning, immune function and inflammation [14,15]. PENK, also termed proenkephalin A 119–159, is a 41 amino acid-long peptide derived as a by-product fragment from the cleavage of a precursor peptide called preproenkephalin A. Apart from PENK, cleavage of this precursor molecule yields several biologically active enkephalins, with leucine-enkephalin (Leu-Enk) and methionine-enkephalin (Met-Enk) being the two main functionally mature forms [15,16,17]. Given the fact that biologically active enkephalins are extremely unstable molecules and difficult to measure in plasma due to their short half-life, PENK has emerged as a surrogate marker of its biologically active counterparts owing to its in vitro stability and long half-life. Elevated levels of ΡΕΝΚ have been associated with various acute and chronic medical conditions, including heart failure, AKI, and sepsis [17,18,19].

The aim of the present study was to investigate the association of point-of-care (POC)-measured PENK with metabolic, inflammatory and renal biomarkers, as well as its prognostic utility in terms of predicting in-hospital mortality in patients presenting to the ED with septic shock.

## 2. Materials and Methods

### 2.1. Population

This prospective observational study was conducted at the ED of Attikon University Hospital, Athens, Greece, a tertiary university hospital that provides all clinical services. Consecutive patients presenting with septic shock to the ED, between May 2022 and May 2023, were enrolled in the study. Septic shock was defined according to the Sepsis-3 clinical criteria proposed in the third international consensus definitions for sepsis and septic shock [1,20] (Supplementary Materials, Table S1). Exclusion criteria were: age less than 18 years, hemorrhagic, refractory or anaphylactic shock, refusal to participate in the study, and chronic administration of corticosteroids. All patients or their next of kin provided written informed consent before enrolment in the study. The study was performed in accordance with the Declaration of Helsinki, and was approved by the institutional review board (IRB) of the Attikon University Hospital (IRB number: ΕΒΔ282/10-05-2022).

### 2.2. Study Design

The following data were obtained from each participating patient: demographics including age and sex; comorbidities; vital signs including respiratory rate (RR), pulse oximetry blood oxygen saturation (SpO2), systolic blood pressure (SBP), diastolic blood pressure (DPB), heart rate (HR) and body temperature; a 12-lead electrocardiogram (ECG); level of consciousness using the Glasgow Coma Scale [21]; laboratory assessments encompassing blood gas analysis, complete blood count, and biochemistry which included urea, creatinine, glucose, serum bilirubin, aspartate aminotransferase (AST), alanine aminotransferase (ALT), high sensitivity troponin T (hs-TnT), C-reactive protein (CRP), procalcitonin, soluble urokinase plasminogen activator receptor (SuPAR), and fibrinogen; estimated glomerular filtration rate (eGFR) using the Modification of Diet in Renal Disease (MDRD) formula [22]; National Early Warning Score (NEWS) [23]; and SOFA score [9].

All routine laboratory testing was performed at the hospital’s central laboratory. Furthermore, a whole blood sample was collected for the measurement of PENK A 119–159 (PenKid), at the patient’s bedside upon arrival at the ED resuscitation room, in the first 30 min upon presentation, using a commercially available point-of-care device (POC), the Nexus IB10 POC (Sphingotec GmbH, Hennigsdorf, Germany), which provides test results via whole blood in 20 min. The Nexus IB10 is an in vitro immunology analyzer that has a built-in centrifugation capability to quantitatively measure analytes in whole blood or plasma samples on a consumable disc [24]. Sphingotest® penKid® is a non-automated immunoluminometric assay (ILMA) for the in vitro diagnostic quantitative measurement of PENK A 119–159 in human ethylenediamine tetraacetic acid (EDTA) plasma [25]. 

After initial management at the ED, patients were admitted either to the intensive care unit (ICU) or to a high dependency unit (HDU), and were followed throughout their length of in-hospital stay, up to the time point of either hospital discharge or death.

### 2.3. Statistical Analysis

Statistical analysis was performed using SPSS version 29.0 (SPSS, Inc., Chicago, IL, USA). Categorical variables are summarized as frequencies and percentages, and quantitative variables are presented as median with interquartile range (IQR). Differences of qualitative parameters between patient subgroups of survivors and non-survivors were tested by using chi-square test, while differences of quantitative parameters were assessed with the use of Mann–Whitney test. Pearson’s or Spearman’s correlation coefficients were used to investigate correlations between normally or non-normally distributed continuous variables, respectively. Due to the highly skewed distribution of values of laboratory variables, as tested by the Kolmogorov–Smirnov test, they were log-transformed for simple correlations and regression analyses. Outcomes examined were the incidence of AKI, defined as an increase in creatinine of ≥0.3 mg/dl or ≥50% within 48 h relative to the initial value, according to the Kidney Disease Improving Global Outcomes (KDIGO) criteria [26] (Supplementary Materials, Table S2), and in-hospital mortality. Receiver operator characteristics (ROC) analysis was used to assess the ability of PENK to predict outcomes. A multivariable logistic regression model with a backward stepwise algorithm was used to investigate independent predictors of in-hospital mortality. A *p* value of <0.05 was considered statistically significant.

## 3. Results

### 3.1. Patient Characteristics

Between May 2022 and May 2023, 61 consecutive patients with septic shock were prospectively enrolled upon presentation to the ED. Patient characteristics are shown in Table 1 and Table 2. Median PENK (IQR) was 205 (129–425) pmol/L. Median Glasgow Coma Scale was 12 (9–15), NEWS Score 12 (10–14), and SOFA Score 6 (4–9). The final diagnoses were: lower respiratory tract infection in 44 (72.1%) patients, urinary tract infection in 10 (16.1%) patients, acute cholecystitis in 2 (3.3%) patients, intra-abdominal infection in 3 (4.9%) patients and of unknown cause in 2 (3.3%) patients.

### 3.2. Correlations

**Figure 1:**
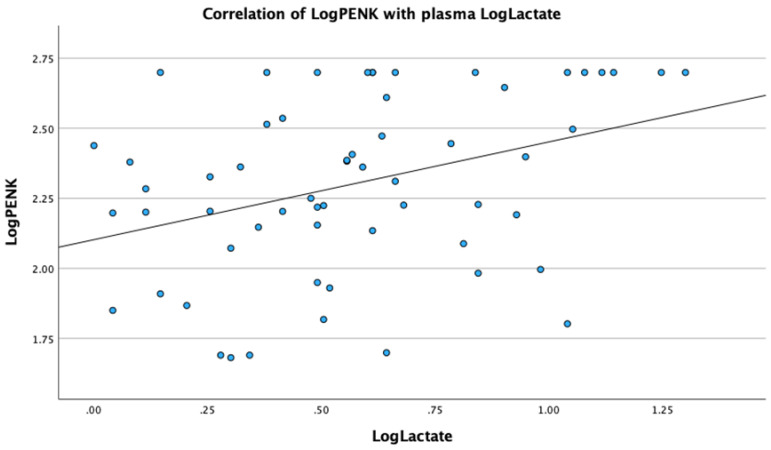
Scatterplot showing the correlation of point-of-care proenkephalin (PENK) with plasma lactate.

**Figure 2:**
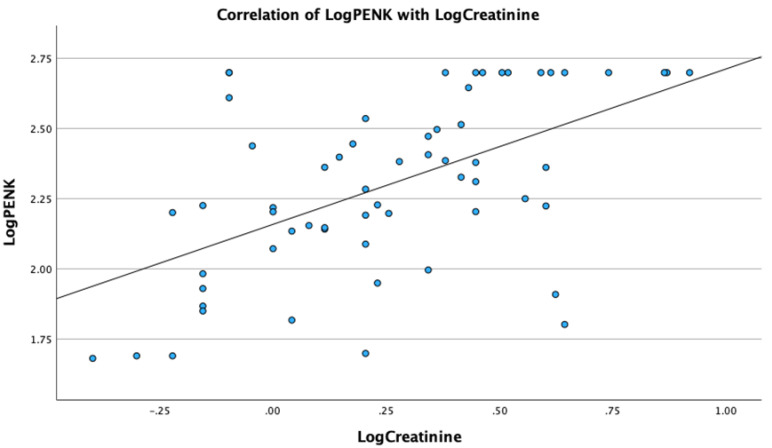
Scatterplot showing the correlation of point-of-care proenkephalin (PENK) with creatinine on admission.

**Figure 3:**
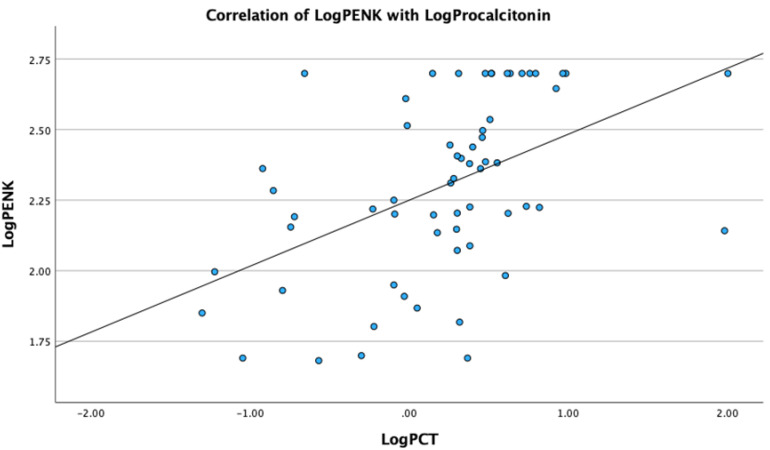
Scatterplot showing the correlation of point-of-care proenkephalin (PENK) with procalcitonin (PCT).

**Figure 4:**
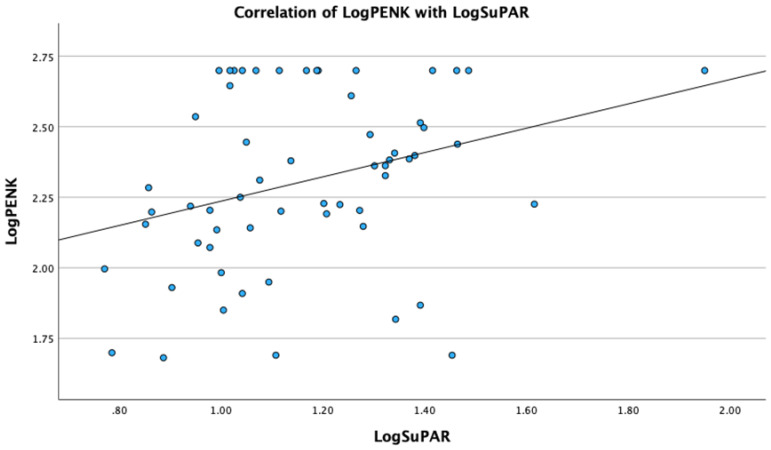
Scatterplot showing the correlation of point-of-care proenkephalin (PENK) with soluble urokinase plasminogen activator receptor (SuPAR).

**Figure 5:**
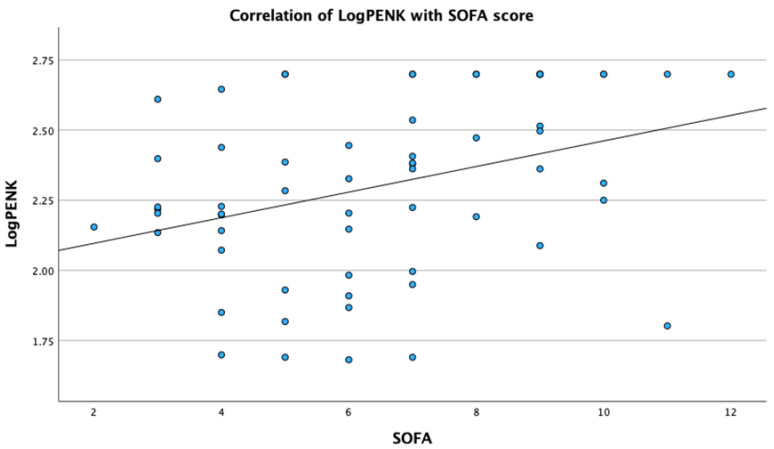
Scatterplot showing the correlation of point-of-care proenkephalin (PENK) with sequential organ failure assessment (SOFA) score.

Analysis revealed significant correlations of PENK with metabolic, renal and sepsis markers. Specifically, LogPENK was positively correlated with LogLactate (rho = 0.369, *p* = 0.004), LogCreatinine (rho = 0.537, *p* < 0.001), LogProcalcitonin (rho = 0.557, *p* < 0.001), and LogSuPAR (rho = 0.327, *p* = 0.011) (Figure 1, Figure 2, Figure 3 and Figure 4). Additionally, a significant positive correlation was observed between PENK and SOFA score, suggestive of the association of PENK with the patient’s clinical severity (rho = 0.391, *p* = 0.002) (Figure 5).

### 3.3. Outcomes

**Figure 6:**
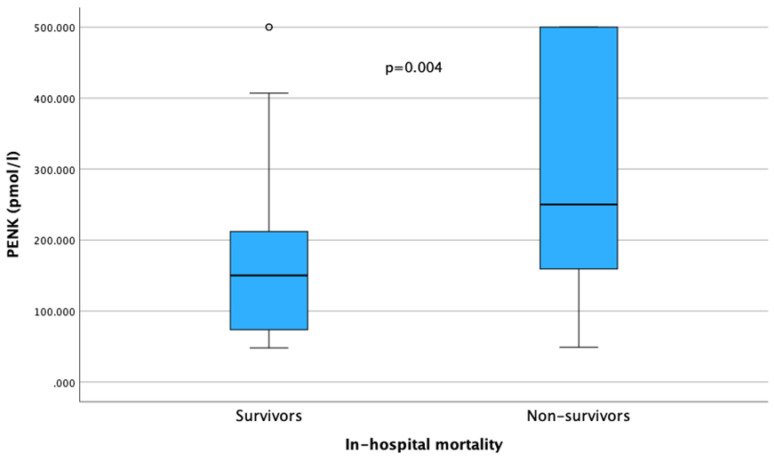
Comparison of point-of-care proenkephalin (PENK) levels between survivors and non-survivors (*p* = 0.004).

During a median (IQR) hospital stay of 6 (2–11) days, 7 patients (12%) developed AKI and 39 (64%) died. POC PENK levels were significantly higher in patients who died [median (IQR), 250 (159–500) pmol/L] compared to survivors [150 (73–220) pmol/L] (*p* = 0.004) (Figure 6).

**Figure 7:**
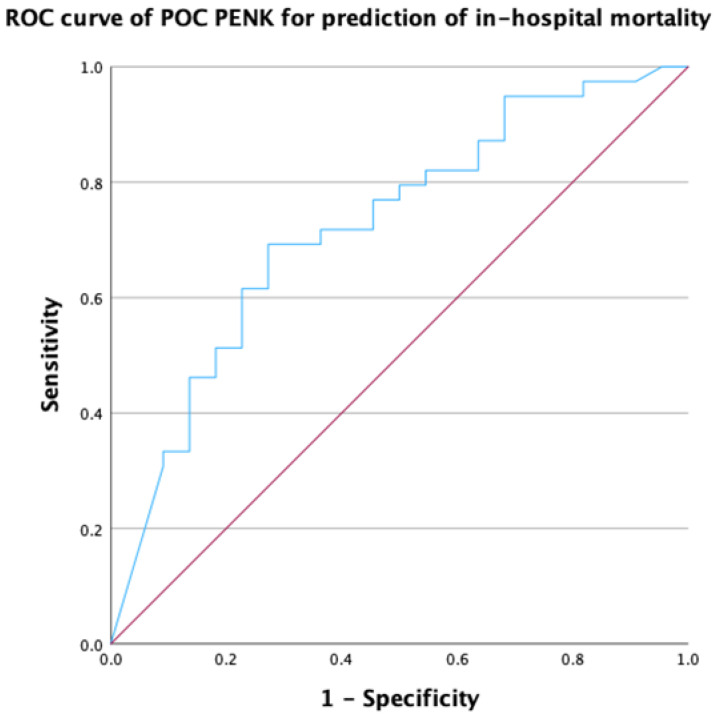
Receiver operating curve (ROC) of point-of-care proenkephalin (PENK) for the prediction of in-hospital mortality. ROC curve is represented by the blue line, whereas the red line corresponds to the diagonal reference line.

POC PENK levels did not differ significantly between patients with versus without AKI during hospitalization (*p* > 0.05). In univariate analysis, age [odds ratio (OR) 1.047, 95% confidence interval (CI): 1.002–1.094, *p* = 0.042], logLactate [OR 6.740, 95% CI:1.055–43.062, *p* = 0.044], and logPENK [OR 15.327, 95% CI:2.214–106.094, *p* = 0.006] were significantly associated with in-hospital mortality. In ROC analysis, POC PENK showed a fair predictive ability for in-hospital mortality, with an area under the curve (AUC) of 0.723 (*p* = 0.004) (Figure 7). A cut-off of 83.1 pmol/l could discriminate survivors from non-survivors with 95% sensitivity and 32% specificity. In a multivariable logistic regression model that included age, SBP, WBC, logCreatinine, logLactate and logPENK, logPENK was the only independent predictor of in-hospital mortality (OR 11.9, 95% CI: 1.7–84.6, *p* = 0.013). When diabetes mellitus and arterial hypertension were added in the model, both diabetes (OR 5.3, 95% CI: 1.0–27.6, *p* = 0.047) and logPENK (OR 12.9, 95% CI: 1.2–136.7, *p* = 0.034) were independently associated with in-hospital mortality, though logPENK remained the strongest predictor.

## 4. Discussion

In this prospective observational study, which included unselected patients presenting to the ED with septic shock, we observed that PENK levels upon presentation were significantly associated with metabolic, inflammatory and renal biomarkers and could independently predict in-hospital mortality.

The median value of PENK in our patient cohort with septic shock was 205 (129–425) pmol/L, which is considerably elevated compared to the median PENK concentration reported in healthy subjects (62.3 pmol/L) [16]. The critical clinical condition of our population, as evidenced by the elevated median SOFA score, which is indicative of severe multi-organ dysfunction, may justify the markedly increased concentration of PENK. Likewise, prior studies in septic patients have shown that PENK levels were associated with the severity of sepsis, and were profoundly elevated in the group of patients with septic shock in comparison to patients with sepsis. These studies have reported various median PENK concentrations in patients with septic shock, ranging from 92 (56–171) pmol/L, *p* = 0.0001 [27], to 118.7 (71.7–245.3) pmol/L, *p* = 0.02 [28], and 205 (87–485) pmol/L, *p* < 0.001 [17]. 

PENK was associated with kidney function, as depicted by a significant correlation of log-transformed baseline PENK values with creatinine levels. This finding is in line with previous studies, in which PENK levels measured in patients with sepsis or septic shock were inversely correlated to creatinine clearance [17] and eGFR [28,29], and positively correlated to creatinine levels [27] and renal SOFA sub-scores [28]. Although serum creatinine is currently used for the assessment of kidney function and the definition and staging of AKI [26], its poor sensitivity and specificity underscore the ever growing need for novel biomarkers that would reflect kidney function in a more precise and timely manner [30].

In septic states, AKI may manifest as a result of perturbations in the renal microvasulature, intrarenal shunting induced by redistribution of blood flow away from the medulla and towards the renal cortex, in combination with focal tubular injury provoked by toxic inflammatory cytokines and oxidative stress leading to apoptosis [31,32]. Recent publications propose the measurement of new early biomarkers of AKI such as Liver Fatty Acid Binding Protein (L-FABP), interleukin-18 (IL-18), Tissue Inhibitor Metal Proteinase-2* Insulin Growth Factor Binding Protein-7 (TIMP2*IGFBP7), Mid-Regional pro-Adrenomedullin (MR-proADM), Kidney Injury Molecule-1 (KIM-1), and Neutrophil Gelatinase-Associated Lipocalin (NGAL) [33,34,35,36]. Further investigations are warranted in order to elucidate the usefulness of each biomarker in the context of sepsis-induced AKI.

Increasing evidence suggests that PENK may serve as a functional biomarker of renal function and kidney injury [15,18]; nevertheless, its appropriateness remains to be further elucidated in large-scale clinical trials. In addition, recent studies have shown that PENK levels may predict AKI in patients with sepsis [17,27,28,29,37,38]. In our population, baseline PENK levels upon ED presentation were not associated with the incidence of AKI. Potential explanations for this observed dissociation between PENK levels and prediction of AKI might be the relatively small patient sample size in our study, which was not powered to examine AKI, coupled with the increased median age in our patient cohort, which might have affected the results, given that most elderly subjects have pre-existing renal decline. Moreover, the high mortality rate within the first 24 h from admission (16/61 patients) eventually resulted in a considerable number of missing values from repeated serum creatinine measurements that would need to be obtained in order to assess AKI in our overall cohort. Additionally, it is worth mentioning a study by Kim et al., which evaluated patients by dividing them into three distinct subgroups, namely suspected sepsis, sepsis and septic shock. In this study, associations between PENK levels and renal function were drawn for each corresponding group of patients. Although PENK levels were positively correlated with the occurrence of AKI, patients with septic shock exhibited elevated PENK levels, regardless of the presence of AKI. In particular, in the subgroup of patients with septic shock, PENK concentrations were 199.7 (101.7–304.3) pmol/L when AKI was present and 117.6 [80.6–209.6] pmol/L in the absence of AKI, yet this difference was not statistically significant (*p* = 0.2128) [29]. Similarly, in the current study, which comprised only patients with septic shock, PENK levels were not significantly associated with the development of AKI.

**Figure 8:**
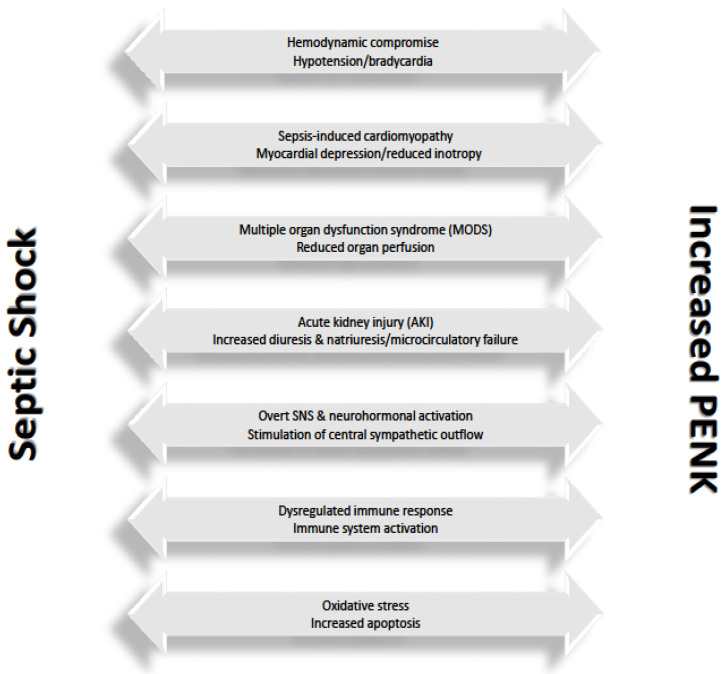
The complex interplay between septic shock and increased proenkephalin levels. Septic shock is characterized by profound hemodynamic instability, which can be further aggravated by the development of sepsis-induced cardiomyopathy [48]. On the other hand, elevated proenkephalin (PENK) levels can lead to further hemodynamic compromise, since it has been shown that PENK induces hypotension and bradycardia, both of which may exert deleterious effects on an already dysregulated cardiovascular system. Decreased blood pressure and heart rate have been attributed to PENK’s inhibitory effect on the catecholaminergic neural stimulation of the heart and the vasculature, resulting in an attenuated response of the heart to adrenergic stimulation, along with peripheral vasodilation through diminished response to sympathetic vasoconstriction. Moreover, increased PENK levels can exacerbate cardiac dysfunction by provoking myocardial depression and causing a direct negative inotropic effect [49,50,51]. Additionally, septic shock can lead to multiple organ dysfunction syndrome (MODS), which can be further mediated by elevated PENK levels via decreased organ perfusion [49,52]. Acute kidney injury (AKI) represents a common finding in patients with septic shock, whereas PENK has been shown to induce natriuresis and diuresis through delta (δ) opioid receptors [25,53]. In this regard, it has been postulated that elevated PENK levels in patients with AKI act as a counter-regulatory mechanism in an attempt to mitigate worsening renal function which, however, becomes maladaptive, eventually leading to untoward alterations in renal excretory function, regional blood flow redistribution, microcirculatory failure and further decline in renal perfusion [17,27,37,54]. Apart from that, a hallmark of septic shock is the overt activation of the sympathetic nervous system (SNS) and the neurohormonal system. In parallel, increased PENK levels have been implicated in the stimulation of the central sympathetic outflow, thus leading to further sympathetic overdrive which, however, may subsequently result in sympathetic exhaustion and downregulation of the sympathetic pathway at the cardiac level [55]. Dysregulated immune response is another key feature of septic shock, while PENK is known to be involved in immunomodulation. Accordingly, elevated PENK levels result in increased release of cytokines by macrophages and T-cells, coupled with marked proliferation of B and T lymphocytes and augmented activation of macrophages, natural killer (NK) cells and neutrophils. The net result is overactivation and exaggerated dysregulation of the immune system [14,56]. Finally, septic shock is governed by tissue hypoxia, leading to cellular dysfunction, mitochondrial dysfunction, metabolic dysregulation and abnormal calcium handling. These abnormalities collectively result in increased oxidative stress and increased production of reactive oxygen species (ROS), which are further aggravated by the proapoptotic effects of increased PENK levels, ultimately leading to a severe compromise of cellular structure and function [46,57]. It should be noted that all of the above represent postulated mechanisms that are potentially implicated in the multifaceted interaction between septic shock and elevated PENK levels.

The aforementioned finding implies that, in the setting of septic shock, other factors, apart from kidney injury, may affect PENK levels. Indeed, in our cohort, baseline PENK levels were also significantly correlated with inflammatory biomarkers, namely procalcitonin and SuPAR, as well as with lactate, which is regarded as a marker of metabolic dysregulation. SuPAR is a glycoprotein mainly expressed on various immune cells and released in the bloodstream upon an inflammatory insult [39]. Elevated levels are considered to reflect the degree of inflammation and are associated with disease severity, while they may also aid in the risk stratification of septic patients presenting to the ED [40,41,42]. In parallel, procalcitonin has been established as a new biomarker for the diagnosis and prognosis of patients with sepsis caused by bacterial infections [43,44]. Regarding the role of lactate in sepsis, hyperlactemia is deemed to be the result of tissue hypoxia combined with overt stress response [45]. Our findings seem to corroborate the notion that elevation of PENK levels in patients with septic shock is multifactorial. In fact, it could be assumed that overactivation of the inflammatory system coupled with hemodynamic compromise and adrenergic overdrive may play a significant role either in the upregulation or even in the decreased clearance of PENK. A possible underlying mechanism observed in an experimental study which, however, warrants extensive testing and further elucidation, is the upregulation of PENK induced by oxidative stress which is known to prevail in septic states and further complicate the multifaceted pathobiology of sepsis [46,47]. The complex interplay between septic shock and increased PENK levels is depicted in Figure 8, where plausible pathophysiological mechanisms that may come into play are being proposed, highlighting the multifactorial interaction between septic shock and PENK.

Importantly, in the current study, PENK levels upon presentation were the strongest predictor of in-hospital mortality (OR 11.9, 95% CI: 1.7–84.6, *p* = 0.013). Similar findings have been reported by previous trials [17,27,28,29,37,38]. PENK levels differed significantly (*p* = 0.004) between survivors [median (IQR), 150 (73–220) pmol/L] and non-survivors [250 (159–500) pmol/L], thus implying that PENK may serve as an important biomarker for the early risk stratification of patients presenting to the ED with septic shock. This finding becomes even more important, considering the fact that the patients in our study had features of severe form of septic shock, as suggested by high SOFA score [58] and high in-hospital mortality (64%, with 26% dying within the first 24 h after admission). Therefore, it could be postulated that even among patients lying in the severe extremes of the septic shock spectrum, PENK measured upon ED presentation may discriminate those with the highest mortality risk. By doing so, POC PENK measurement upon ED arrival may substantially aid in the decision-making process with regard to the provision of appropriate type of treatment, level of monitoring and patient allocation. Although patient mortality in our study was strikingly high (64%), when compared to previously published studies examining the prognostic utility of PENK in patients with sepsis, it has to be emphasized that we recruited unselected elderly patients presenting to the ED exclusively with septic shock, and we measured in-hospital mortality, contrary to prior studies that measured 7-day [17], 30-day [28,29,37,38] and 90-day [27] mortality in patients recruited in the ED [17,28,38] and the ICU [27,28,29,37]. Certain baseline patient characteristics, such as the high median age (83 years) and the presence of multiple comorbidities, coupled with a high SOFA score [58], may in part explain both the high in-hospital mortality in the overall population and the notable percentage of patients (26%) who died within the first 24 h from ED admission (comprising 41% of the total deaths).

An important strength of this study is that PENK levels were measured upon presentation to the ED at the patient’s bedside by means of a point-of-care device, using whole blood sample. This measurement method of POC PENK levels yielded statistically significant results regarding survival prediction. Our study points towards the fact that previously reported results regarding the prognostic role of PENK levels, from studies utilizing frozen plasma [17,27,28,29,37,38] of patients with septic shock, are reproducible with a point of care device in the ED. This is of major importance, considering the fact that the initial resuscitation of patients with septic shock takes place in the ED, where readily available PENK levels on-site may provide additional information during ongoing resuscitation. The recent literature suggests that patients with sepsis may face significant admission delays to the ICU, a phenomenon that inflicts an additional mortality burden in the already compromised subgroup of patients with septic shock [59]. Risk stratification, provided by a single PENK value upon ED presentation, may identify a subset of patients who carry the poorest prognosis, and may thus benefit from the implementation of an aggressive therapy protocol in combination with close monitoring and early ICU admission.

### Limitations

Undoubtedly, the small population of our study is a significant limitation, yet the current findings may serve as a boost for further research on the role of PENK in septic shock states, as well as on the pathophysiological pathways responsible for its increase in patients with septic shock. More importantly, our survey suggests that PENK levels may distinguish survivors from non-survivors; this may actually act as an impetus for future studies to further focus on determining a cut-off level that would discriminate patients with dismal survival from those with favorable prognosis. Moreover, we examined the potential utility of a single measurement of PENK upon patient presentation to the ED that could aid in the risk stratification of patients with septic shock. Future studies could further evaluate the prognostic importance of changes in PENK levels after admission. 

## 5. Conclusions

In conclusion, POC PENK measured upon presentation to the ED may reliably and independently predict poor survival among patients with septic shock in a timely manner. The findings of this study highlight an emerging prognostic role of PENK as a promising POC biomarker in the ED setting, which may facilitate in the early identification of subjects with septic shock who require close monitoring and intensive care management.

## Figures and Tables

**Table 1:** Clinical characteristics of study patients.

	All Patients (*n* = 61)	Survivors (*n* = 22)	Non-Survivors (*n* = 39)	*p*-Value
Age, years	83 (71–88)	77 (66–87)	84 (76–88)	0.103
Sex (female), n (%)	32 (53)	9 (41)	23 (59)	0.175
***Vital signs***				
Temperature, °C	37.8 (36.7–38.3)	37.8 (36.4–38.0)	37.9 (36.8–38.5)	0.265
SBP, mmHg	82 (71–90)	84 (73–90)	81 (70–90)	0.707
DBP, mmHg	50 (41–56)	50 (42–60)	48 (40–55)	0.313
Heart rate (HR), beats per minute (bpm)	110 (80–129)	111 (82–122)	109 (75–130)	0.952
Respiratory rate (RR), breaths per minute	22 (20–25)	22 (20–24)	24 (20–25)	0.358
Pulse oximetry oxygen saturation (SpO2), %	94 (89–98)	91 (88–96)	96 (90–98)	0.100
***Comorbidities***				
Arterial hypertension, *n* (%)	39 (64)	12 (55)	27 (69)	0.251
Coronary artery disease, *n* (%)	8 (13)	2 (9)	6 (16)	0.462
PCI/CABG, *n* (%)	3 (5)	1 (5)	2 (5)	0.919
Atrial fibrillation, *n* (%)	24 (39)	8 (36)	16 (41)	0.720
Heart failure, *n* (%)	9 (15)	2 (9)	7 (18)	0.349
Permanent pacemaker, *n* (%)	3 (5)	0	3 (8)	0.182
Diabetes mellitus, *n* (%)	23 (38)	7 (32)	16 (41)	0.476
COPD, *n* (%)	13 (21)	6 (27)	7 (18)	0.393
Prior stroke, *n* (%)	8 (13)	3 (14)	5 (13)	0.928
Chronic renal dysfunction, *n* (%)	3 (5)	2 (9)	1 (3)	0.258
Thyroid disease, *n* (%)	12 (20)	7 (32)	5 (13)	0.073
Dementia, *n* (%)	17 (28)	4 (18)	13 (33)	0.205
Sleep apnea syndrome, *n* (%)	1 (2)	1 (5)	0	0.179
***Management***				
ICU admission, *n* (%)	7 (12)	3 (14)	4 (10)	0.691
***Clinical risk scores***				
GCS	12 (9–15)	13.5 (9.75–15)	10 (7–14)	0.012
SOFA	6 (4–8.5)	4.5 (3.75–6.25)	7 (5–9)	0.003
NEWS	12 (10–13.5)	11.5 (10–12.25)	12 (9–14)	0.468
***Length of stay (LOS)***				
Total LOS, days	6 (2–11)	11 (6–14)	3 (1–8)	<0.001
***In-hospital mortality***, *n* (%)	39 (64)	NA	NA	

Abbreviations: CABG = coronary artery bypass grafting; COPD = chronic obstructive pulmonary disease; DBP = diastolic blood pressure; GCS = Glasgow Coma Scale; ICU = intensive care unit; NA = not applicable; NEWS = National Early Warning Score; PCI = percutaneous coronary intervention; SBP = systolic blood pressure; SOFA = Sequential Organ Failure Assessment.

**Table 2:** Laboratory parameters of study patients upon presentation to the Emergency Department.

	Normal Values	All Patients (*n* = 61)	Survivors (*n* = 22)	Non-Survivors (*n* = 39)	*p*-Value
***Arterial blood gas analysis***					
pH	7.350–7.450	7.31 (7.22–7.41)	7.31 (7.22–7.42)	7.31 (7.20–7.41)	0.721
PCO_2_, mmHg	32.0–48.0	39 (31–51)	43 (32–57)	38 (30–48)	0.309
HCO_3_, mEq/L	22.0–26.0	20 (15–26)	22 (17–28)	19 (15–26)	0.316
Lactate, mmol/L	1.0–1.8	3.6 (2.1–6.8)	3.1 (1.7–4.4)	3.9 (2.6–8)	0.054
***Laboratory analysis***					
Hemoglobin, g/dL	13.5–17.5	11.3 (9.5–13.3)	11.9 (9.6–14.1)	10.9 (9.3–13)	0.223
WBC, ×10^3^/μL	4.00–11.00	14.04 (9.55–21.54)	13.62 (8.8–26.22)	14.92 (10.73–21.14)	0.975
PLT, ×10^3^/μL	150–400	208 (144–329)	204 (151–327)	217 (142–338)	0.866
Urea, mg/dL	16.6–48.5	98 (51–152)	80 (40–113)	109 (63–176)	0.054
Creatinine on admission, mg/dL	0.5–0.9	1.7 (1.0–2.9)	1.5 (0.8–2.5)	2.2 (1.1–3.3)	0.133
Creatinine at 48 h, mg/dL		1.1 (0.8–2.3)	1.0 (0.6–1.8)	1.2 (0.8–2.6)	0.212
Glucose, mg/dL	74–106	155 (102–178)	123 (96–175)	158 (110–179)	0.190
AST, U/L	<32	31 (17–49)	17 (13–34)	33 (25–59)	0.007
ALT, U/L	<33	19 (11–31)	12 (9–26)	23 (12–31)	0.038
CRP, mg/L	0.00–6.00	122 (69–185)	134 (36–196)	119 (79–171)	0.794
Hs-TnT, pg/mL	<14	78 (48–146)	76 (38–132)	80 (52–150)	0.335
Procalcitonin (PCT), ng/mL	<0.5	2.06 (0.84–3.49)	1.41 (0.57–2.43)	2.60 (1.59–4.13)	0.036
SuPAR, ng/mL	<4	13.1 (10.0–21.4)	11.4 (8.2–24)	13.4 (10.6–20.4)	0.481
Proenkephalin (PENK), pmol/L	<80	205 (129–425)	150 (73–220)	250 (159–500)	0.004
Fibrinogen, mg/dL	200–400	404 (340–455)	406 (318–467)	401 (358–452)	0.859
***Electrocardiogram***					
Sinus rhythm, n (%)		45 (74)	17 (77)	28 (72)	0.640
Atrial fibrillation, n (%)		16 (26)	5 (23)	11 (28)	
***Left Ventricular Ejection Fraction***					
<40%, n (%)		10 (16)	2 (9)	8 (21)	0.247
≥40%, n (%)		51 (84)	20 (91)	31 (79)	

Abbreviations: ALT = alanine aminotransferase; AST = aspartate aminotransferase; CRP = C-reactive protein; HCO3 = bicarbonate; Hs-TnT = high sensitivity troponin T; PCO2 = partial pressure of carbon dioxide; PLT = platelet count; SuPAR = soluble urokinase plasminogen activator receptor; WBC = white blood cell count.

## Data Availability

Data are contained within article.

## Supplementary Materials

The following supporting information can be downloaded at: https://www.mdpi.com/article/10.3390/biomedicines12051004/s1. Table S1: Sepsis and septic shock clinical criteria according to the Third International Consensus Definitions for Sepsis and Septic Shock, modified from [1]; Table S2: Proposed staging for acute kidney injury according to KDIGO criteria, modified from [26].
