# Supplementary material for: Point-of-Care Serum Proenkephalin as an Early Predictor of Mortality in Patients Presenting to the Emergency Department with Septic Shock
Source: Biomedicines. 2024 May 2;12(5):1004. doi: 10.3390/biomedicines12051004 (PMC11117930; doi:10.3390/biomedicines12051004)
Supplement: Supplementary file 1 [file biomedicines-12-01004-s001.zip › biomedicines-2965514-supplementary.pdf]

SUPPLEMENTARY MATERIAL

Table S1. Sepsis and septic shock clinical criteria according to the Third International Consensus Definitions for Sepsis and Septic Shock, modified from [1] :

	Sepsis	*Septic shock*
2015 Clinical criteria	Suspected or documented infection and an acute increase of ≥2 SOFA points (a proxy for organ dysfunction)	Sepsis and vasopressor therapy needed to elevate MAP ≥65 mm Hg and lactate >2 mmol/L (18 mg/dL) despite adequate fluid resuscitation

Abbreviations: MAP=Mean Arterial Pressure; SOFA Sequential Organ Failure Assessment

Table S2. Proposed staging for acute kidney injury according to KDIGO criteria, modified from [26]:

**STAGE**	*Serum Creatinine*	*Urine output*
1	1.5–1.9 times baseline or ≥0.3 mg/dl (≥26.5 mol/l) increase	<0.5 ml/kg/h for 6–12 h
2	2.0–2.9 times baseline	<0.5 ml/kg/h for ≥12 h
3	3 times baseline or ≥4.0 mg/dl (≥353.6 mol/l) increase or initiation of RRT or in patients	<0.3 ml/kg/h for ≥24 h or anuria ≥12 h

Abbreviations: RRT=Renal Replacement Therapy
